# Rosalind’s Ghost: Biology, Collaboration, and the Female

**DOI:** 10.1371/journal.pbio.2001003

**Published:** 2016-11-04

**Authors:** Caroline Wagner

**Affiliations:** Ohio State University, Columbus, Ohio, United States of America

Anyone familiar with the history of genomic science will know the contribution of, and controversy about, Rosalind E. Franklin. No one disputes that her work in X-ray diffraction imaging, resulting in Photo 51, was foundational to the discovery of the double helix. The controversy arises in how well or poorly Franklin worked as a team member with Watson, Crick, and Wilkins, and they with her. Some interpretations of the story appear to reveal aggressive gender segregation on the part of some colleagues. Rosalind’s ghost may be uneasy with recent findings about genomic collaborations.

Over the decades, science has become increasingly collaborative and team based. As measured by coauthorships on refereed scientific papers, the number of multi-authored papers grew slowly in the postwar decades but took a sharp turn upwards in 1990. Growth is fastest at the international level. The count of authors per paper has also grown. Coauthorships are now the norm in science. These multi-authored papers tend to attract more citations. The more countries listed in the address fields, the greater the citation impact of an article in the science, technology, engineering, and mathematics (STEM) fields.

Many scholars have documented these changes, parsing the growth by field, by nation and region, by citation, and by social dynamics. Focus is often placed on social practices of teams and epistemic communities. Disciplines, university practices, and faculties are examined. Depending upon the perspective of the inquirer (e.g., quantitative/macro, qualitative/micro), the changes in science dynamics are attributed to diverse drivers, such as broadening economic development, the information revolution, ease of transport, political shifts, academic competition, and improving education levels. Reasons offered for the dramatic changes in research practices remain unsatisfying.

One factor remains fairly constant: women are underrepresented in terms of authorships, including first and/or last authorships (whichever is more prestigious), coauthorships, and in the granting of scientific prizes (see Fig 1). Although their ranks are growing over time (see Fig 2), women scientists receive less grant funding, and their published work is cited less frequently by other scientists (even by other women). Globally, women account for fewer than 30% of fractionalized authorships [1]. As a *PLOS ONE* article just showed, women are underrepresented on journal editorial boards [2].

**Fig 1:**
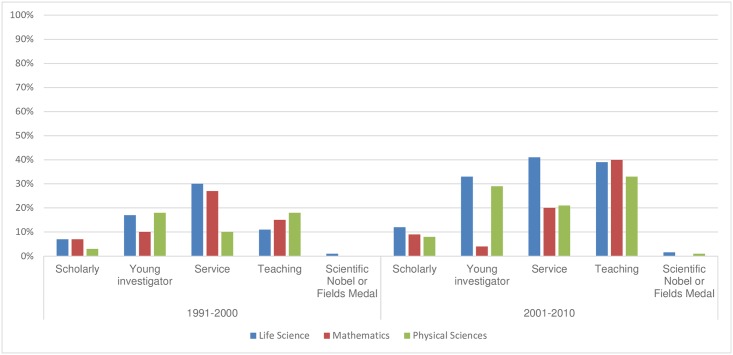
Percentage of female winners by STEM award type and field, 1991–2010 [3].

**Fig 2:**
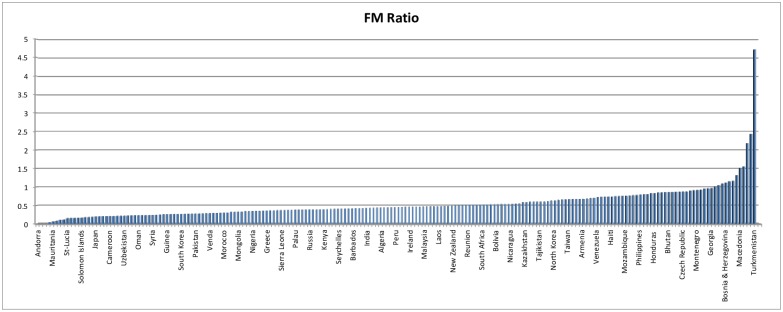
Ratio of female to male authorships for top 30 scientifically advanced countries, 2008–2013 [1].

Overall, the more elite the scientist, the more likely they are to work at the international level; however, female collaborators are less likely to be working internationally and are more likely to collaborate locally. This means that they are also less likely to coauthor with top scholars. Among countries (as one might expect), some are much more likely to have women scientists authoring papers than others (see Fig 3). (There is a notable lack of correlation between those countries ranking higher on United Nations Human Development gender equality index and those with greater scientific “equality.” Portugal and Poland stand out with high female participation in scientific publishing.) But, even for what are considered more “egalitarian nations,” publication patterns are dominated by men. Women publish fewer articles than men and, on average, have shorter careers. The shortened career is sometimes pointed to as contributing to the persistence of gender gaps, but even accounting for the shortened career, women’s publications do not receive proportionate attention in terms of citations.

**Fig 3:**
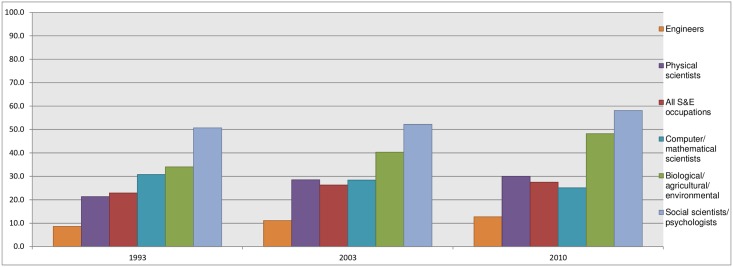
Women as a percentage of all science and engineering workers by fields in the United States, 1993–2010 [4].

Zeng et al. [5] add to this literature by examining collaboration patterns across disciplines, career stage, and gender. They find that, for six fields, women differ from men in their propensity to collaborate. Across the fields studied, women had significantly fewer coauthors. This finding, in itself, should not raise an alarm, because Wagner et al. [6] found that Nobel Prize winners (the vast majority of whom are male) in medicine also have fewer coauthors in their careers. That fact, in itself, is not a career killer. Nevertheless, in the details, Zeng et al. show that women have a lower number of distinct coauthors, which could be a problem in that new collaborations often result in more creative outputs. They further show that women participate less frequently in collaborations, which is unexpected because cultural memes suggest that women are better at cooperating, which is supported by some research [7].

Perhaps the least expected of Zeng et al.’s findings are those in the biological sciences. These fields have been attracting more women than other STEM fields, as Fig 3 shows. Indeed, the U.S. Census reports that women earn more than 50% of all degrees in the life sciences. Zeng et al. find the gap to be so great, with such a low participation of women, that it appears to constitute “gender segregation.” The number of women coauthoring in genomics is so far below expectation that chance or shorter careers cannot be the reason for the disparity.

Perhaps the most enigmatic finding from Zeng et al. is that women do not repeat coauthorships as frequently as male counterparts. On one hand, this might indicate practices related to active search for new ideas, as the authors note: “….[w]e find evidence for the hypothesis that female scientists are more open to novel collaborations…” Furthermore, this resonates with our work on Nobel Laureates, in which the communication networks suggested that prize winners were more likely than elite peers to reach outside of their immediate networks to seek new ideas. However, it is clear from their data that this characteristic of women’s teams is not translating into citations, publications, and certainly not into scientific prizes.

Zeng et al. focus on those female academics who persisted in their careers—a fact, they note, that must be put into context, because a good deal of evidence suggests that women “leak” out of the educational and professional system at rates higher than men [8]. In addition, women in STEM careers (especially those women with children) are much more likely than men to leave academic careers before being granted tenure. Given the documented bias in favor of men in academic hiring decisions, in salary, in promotion and tenure decisions, and even in reviews of teaching by students, it is mystifying how the academy can continue to ask why they have a problem attracting and retaining women in STEM fields. The women in Zeng et al.’s study have persisted beyond other obstacles and disincentives, and so they may be operating according to learned mechanisms for achievement that are difficult to decode.

The findings support earlier work and raise new questions. Why would women scientists behave differently from their male counterparts? Zuckerman and Cole [9] asked similar questions in 1975, suggesting a “Triple Penalty” influencing women in science. Of the three penalties, only one continues to be noted in the literature: that women face actual discrimination in the scientific workforce. The other two—that it is culturally inappropriate for women to seek careers in science and that women scientists view themselves as less competent—have been the object of such a tsunami of social conditioning that no one dares to ask if they may continue to operate. A fourth “penalty” has been added in the literature: unconscious bias.

Handelsman et al. [10] detail statistics about women in science, policy actions to address the obstacles, and the impact of “unconscious bias”–concepts that emerged from ADVANCE Institutional Transformation Program funded by the U.S. National Science Foundation (NSF). ADVANCE projects were funded to analyze the environment for women scientists and to explore interventions that might improve that environment. In a series of studies, scholars found that most people were not aware of holding discriminatory views, but that decisions (especially those made under stress) would sometimes reveal a gender bias (held by men and women). Trainings were developed. One program, called ADEPT (Awareness of Decisions in Evaluating Promotion and Tenure), was made widely available to research universities. The University of Wisconsin reports seeing positive changes as a result of implementing workshops aimed at ameliorating unconscious bias. Even so, Easterly and Ricard [11] find that “[i]nstitutions of higher education today remain gendered institutions, with males holding the majority of professorships and upper administrative positions, such as president and provost….” p. 63. Certainly, this is the case at my own university.

Despite the raised awareness from ADVANCE and social conditioning, Zeng et al. show that many of the characteristics of the careers of women scientists have changed little in the 40 years since Zuckerman and Cole wrote about women in science. Women still hold lower ranks than men. They still have fewer citations. They publish fewer articles. And, apparently, they are “less fully integrated into the scientific community within their fields of specialization, thus reducing the probability of carrying on useful scientific inquiry….” (p. 93), as Zuckerman and Cole noted four decades ago. Moss-Racusin et al. [12] found that actual discrimination is practiced by men and women. From somewhere in the Heaviside layer, Rosalind Franklin already knows this fact.

Just as in the study of collaboration as a whole, a problem with this (and many other assessments of gender) is the orientation toward the individual actor—such as the PhD, the postdoc, or the faculty member—rather than on the systemic domain, which data suggest is where the problem lies. Examining the individual ant does not explain the anthill. Viewing the actions of women in collaborations, publications, and citations does not reveal the reasons for the continued disparities between men and women in science. Many articles reviewed for this primer (full bibliography available on figshare [13]) talked about the potential benefit of having more women fully contribute to scientific research—as fully as men. Yet after many years, disparities stubbornly persist. New methods in social science modeling, such as those being conducted by Kathleen Carley [14], could potentially build agent-based models with various of these assumptions to see which are more determinant of unequal outcomes, and attention can be paid there.

Perhaps deep in our collective genome there is some instruction to “treat females differently.” As Joseph Campbell pointed out, “woman is life, and man is the servant of life…” Perhaps that instruction has been foundational to our species’ survival. The opposite may now be true: treating women the same is essential to our survival. Just as many of our other learned behaviors make social life possible, so treating the work of women scientists as equally worthy of consideration will not only make social, academic, and intellectual life possible; it offers the possibility of improving our species’ chances of long-term survival. It requires that people consciously choose to seek out, honor, and support the work of women. Perhaps then, Rosalind’s ghost can rest.

## Abbreviations

ADEPT: Awareness of Decisions in Evaluating Promotion and Tenure
NSF: National Science Foundation
STEM: science, technology, engineering, and mathematics
